# Equity and trends in general practitioners’ allocation in China: based on ten years of data from 2012 to 2021

**DOI:** 10.1186/s12960-023-00841-5

**Published:** 2023-08-02

**Authors:** Ruxin Kou, Kangni Mei, Yuqing Bi, Jingwen Huang, Shilan Yang, Kexuan Chen, Wei Li

**Affiliations:** School of Public Health, Weifang Medical University, Weifang, 261021 Shandong China

**Keywords:** General practitioner, Equity, Allocation, Prediction

## Abstract

**Background:**

General practitioners (GP) are the gatekeepers of residents' health, 2021 is the 10th year of the establishment of the GP system in China. This study aims to assess the equity and trends of GP allocation in China from 2012 to 2021, summarize the efforts and progress of GPs in China during the decade, predict the development trend of GPs in mainland China in the next 5 years to provide a reference for regional health planning and rational allocation of GPs in China.

**Methods:**

Data from 2012 to 2021 on GPs in 22 provinces, 5 autonomous regions, and 4 municipalities directly under the central government in mainland China (excluding Hong Kong, Macao, and Taiwan) are collected by us. Gini coefficient, Lorenz curve and health resource agglomeration degree (HRAD) were used to analyze the equity of the allocation of GPs in China from different dimensions, a Grey prediction model was used to forecast the number of GPs in 2022–2026.

**Results:**

The number of GPs in mainland China increased from 109 794 to 434 868 from 2012 to 2021, with 3.08 GPs per 10 000 people in 2021. The Gini coefficient of GPs allocation by population in China decreased from 0.312 to 0.147 from 2012 to 2021, while the Gini coefficient of geographic dimension remained between 0.700 and 0.750. Compared with the degree of curvature of the Lorenz curve in the geographic dimension, the degree of curvature of the population and economic dimension were smaller. In 2021, the HRAD in the Eastern region was 4.618, the Central region was 1.493, with different degrees of imbalance among regions, the HRAD/PAD (population agglomeration degree) in the Eastern, Central and Western regions were 1.196, 0.880 and 0.821, respectively. GPs in the Eastern region is still concentrated, while the Central and Western regions were at a similar level, GPs were more scarce. The GM (1,1) model predicts that the number of GPs in mainland China will reach about 720 000 in 2026, the number of GPs per 10 000 people will reach 4.9.

**Conclusion:**

After a decade of development, the number of GPs in China has increased significantly. It has reached the goal of the GP system when it was first established. However, the equity of the geographical dimension, both in terms of Gini coefficient and HRAD, has great differences between different regions. The average Gini coefficient at the geographic dimension is 0.723. The average HRAD index was 4.969 in the East and 0.293 in the West. The Western region has the problem of insufficient GP allocation in both population and geographical dimension. In the future, the number of GPs in China will continue to grow rapidly with the support of policies. The “2030” goal, proposed in 2018, is expected to be achieved by 2026. Due to certain factors (such as COVID-19), the actual situation may be different from the predicted results.

**Supplementary Information:**

The online version contains supplementary material available at 10.1186/s12960-023-00841-5.

## Background

The World Organization of family doctors defines a general practitioner (GP) as “a person who provides comprehensive health care services to everyone who seeks health care, if necessary, arranges for additional health service personnel to provide related services to him or her [1]”. Some experts have also suggested that “any national health care system that is not based on well-trained GPs is doomed to pay a high price [2]”. GPs are highly integrated compound clinicians. They mainly undertake integrated services such as diagnosis, treatment and referral of common diseases, prevention and health care, patient rehabilitation, chronic disease management and health management at the grassroots level. They were known as the “gatekeepers” of residents’ health [3].

The concept of general medicine was introduced to mainland China in the late 1980s [4]. Before that, there was a three-tier system of medical services in China: county hospitals, township health centers and “barefoot doctors (rural informal medical workers) [5]”. Traditional Chinese medicine, likewise occupies a major part of the medical profession in China [6]. Since the introduction of the concept of general practice in China, the first training center for Chinese general practitioners was established in 1989 at the Capital Medical University in Beijing. It began to spread and promote the concept of general medicine throughout the country [4]. The emergence of general practitioners has gradually replaced most of the “barefoot doctors” and a significant proportion of Chinese medicine practitioners, promoted the development of modern medicine [7]. In 2011, The State Council issued “the Decision of The State Council on Establishing General Practitioners [8]”. The importance and necessity of establishing a GP system was highlighted, a series of top-level designs were developed around the training model, the practice model, the use of incentives for GPs, and the GP system was formally established [9]. The document also proposes to accelerate the growth of the GP workforce, with the aim of having essentially two to three qualified GPs for every 10 000 residents in urban and rural areas by 2020. Since 2011, the government has introduced a series of policies and measures to promote the development of GPs in China. With the joint efforts of many parties, the GPs in China have experienced a period of rapid and stable development, finally reached the present scale [10, 11].

Health equity refers to the fact that people have equal access to basic health care services according to their health needs and is one of the core indicators used to evaluate national health systems, especially primary health care systems. The allocation of health resources (this paper refers to the number of GPs) is a basic index to evaluate health equity [12]. One of the most prominent problems facing China's health system is the unreasonable allocation and unbalanced geographical distribution of health resources [13]. Therefore, it is necessary to analyze the unevenness of regional distribution and predict the development trend, so as to provide reference for the formulation of health policy [14]. Due to the late start of general family medicine and the short time of implementation of the GP system in China, the number of GPs is still insufficient and their skill level is relatively low [15]. Although the number of GPs is continuously increasing, the increase in the supply of health workers does not necessarily lead to a decrease in the inequality of distribution [16].

A number of studies have assessed inequalities in national and international GP. For example, Theodorakis [17] plotted a Lorenz curve and calculated the Gini, Atkinson, Robin Hood indices and decile ratios to investigate the level of inequality among GPs in Albania; Toyabe [18] used the Gini, Atkinson and Theil indices as indicators of the unequal distribution of GPs in the population, demonstrated that after the implementation of the new system of postgraduate internship, the regional uneven distribution will be aggravated. Many domestic scholars have also studied GP resource allocation analysis [19, 20]. In these studies, the main methods used by researchers to measure equity include the Gini coefficient, the Theil index, the concentration index, the Lorenz curve, among others. Each method and index has its own advantages and disadvantages, the conditions under which they apply are different. The Lorenz curve was proposed by Austrian statistician M.O. Lorenz (Max Otto Lorenz, 1876–1959) in 1907. The Gini coefficient was introduced by the Italian economist Corrado Gini in 1912 and first used by Hirschman in 1945, when he used statistical methods to calculate the distribution of social income in several countries. Health resource agglomeration degree (HRAD) refers to the proportion of total health resources gathered in a certain area with 1% of the total land area of the measured area [19]. Population resource aggregation degree (PAD) is a measure of GP resource allocation from a population perspective. Compared to the Gini coefficient, HRAD and PAD, when used together, can show whether local health care resources meeting the needs of local residents [21].

The Grey prediction model was proposed by Chinese scholar, Professor Julong Deng, in 1982 as an innovative solution to the problems of scientific research such as unsatisfactory calculation results due to the small amount of data, frequent information, poor regularity of data [22]. It can scientifically predict the trend and situation of a certain period of time in the future under the uncertainty of small sample information and information systems. In particular, it outperforms other prediction models in short-term prediction. This model has the advantages of simplicity, ease of learning, high prediction accuracy, in addition to having no special requirements on sample size, data type, and distribution. In terms of forecasting, the forecasting range of health manpower forecasting is usually 10 and 15 years [23]. This is consistent with the results of studies on the accuracy of population forecasting citations available [24–26].

Our study aims to analyze the current status, equity, trends of GPs allocation in China from 2012 to 2021. The equity of resource allocation can be vividly reflected by using the Lorenz curve and the Gini coefficient together. Therefore, in this paper, we use the Lorenz curve and the Gini coefficient to analyze the equity of GP allocation in mainland China. Based on this, the HRAD proposed in this study considers the equity of resource allocation in terms of population distribution and geographical distribution, analysis regional equity differences. Thus, HRAD has been applied to assess equity in different regions in terms of both geographic and demographic aspects. Finally, a GM (1,1) model was used to forecast the human resource development of GPs in mainland China from 2022 to 2026. The article provides a predictive analysis for achieving the “2030” target, provides a reference for improving the policies related to GPs in China, also provides theoretical support for the development of GPs in China.

The rest of the paper is organized as follows: section “Methods” indicates the data sources, presents the methodology and predictive models for equity evaluation; section “Results” introduces the analysis of the results after data processing by the analytical method, describes the prediction results of the forecast model; Based on the analytical results in section “Results”, Section “Discussion” provides a comprehensive discussion and analysis of GPs in mainland China from 2012 to 2021, pointing out the strengths and problems of GPs in China and making some recommendations; Section “Conclusion” summarizes the whole text, providing an outlook on the development of general medicine in China.

## Methods

### Data source

The data of GPs in 31 provinces, autonomous regions and municipalities directly under the Central government (excluding Taiwan, Hong Kong and Macao) from 2012 to 2021 were used as research materials, the number of GPs in this study refers to the total number of GPs (assistants) who have registered as GPs or who have obtained the training certificate of GPs. The required data was obtained from the 2013–2022 China Health Statistical Yearbook [27–36]. Data for the total population of each region at the end of the year were from the China Statistical Yearbook for the years 2013 to 2022 [37–46]. The division of health regions was based on the China Health and Health Statistical Yearbook. Eastern, Central and Western region division standards: The Eastern region includes 11 provinces and municipalities directly under the central government, including Beijing, Tianjin, Hebei, Liaoning, Shanghai, Jiangsu, Zhejiang, Fujian, Shandong, Guangdong, Hainan; the Central region comprises eight provinces, including Shanxi, Jilin, Heilongjiang, Anhui, Jiangxi, Henan, Hubei, Hunan; the Western region includes Inner Mongolia, Chongqing, Guangxi, Sichuan, Guizhou, Yunnan, Tibet, Shaanxi, Gansu, Qinghai, Ningxia, Xinjiang, 12 provinces, autonomous regions and municipalities under the central government.

### Evaluation methods

#### Gini coefficient and Lorenz curve

The Lorenz curve is a curve used to reflect the equity of resource allocation, mainly based on the arc of the curve to reflect the equity level. The greater the arc of the line, the worse its equity level [47]. In this paper, it means the curve formed by the number of GPs in each region as a percentage of the total number of GPs within a country and its corresponding population/economy/geographic area as a percentage of the total population/economy/geographic area [48]. Specifically, in the two-dimensional plane, the line formed by the cumulative percentage of population/economy/geographic area as the horizontal coordinate and the cumulative percentage of the number of GPs in each region as the vertical coordinate, respectively, the corresponding scattered points are called the Lorenz curve. The geographic and demographic dimensions are easily understood because most researchers work from these two perspectives. As for the economic dimension, we used the regional gross domestic product (GDP) to represent the economic development of a region. This allows us to explore the situation in terms of GP resource allocation from an economic perspective. In a Lorenz curve, the diagonal from the origin of the coordinate to the vertex in the upper right corner of the square is called the line of absolute equality and does not exist in general. The actual Lorenz curves were all below the line of absolute equality [47].

The Gini coefficient is one of the indicators used to quantitatively evaluate the equity of resource allocation [49]. Mr. Zhang Jianhua of Shanxi Agricultural University in China later summarized a simpler formula: the percentage composition of GPs in each region of China is arranged from smallest to largest, the cumulative percentage of population/economy/geographic area of each region were divided into n groups. Assuming that the cumulative percentage share of groups 1 to i is Yi, then the Lorenz curve will pass through the point (i, Yi). Defining Y0 = 0 and Yn = 1 will give us i = 0, 1, 2, … n, on the Lorenz curve, for a total of n + 1 points. Then use the trapezoidal rule to integrate the area under the Lorenz curve and find the area of the right triangle, which eventually leads to the Gini coefficient = A/(A + B) = 1−2B. The Gini coefficient takes values from 0 to 1, with values closer to 0 indicating better fairness; in general, its value below 0.2 implies absolute mean, greater than 0.5 indicates highly unfair [50].

#### Agglomeration degree

Agglomeration degree is generally necessary to consider both health resource agglomeration degree (HRAD) and population agglomeration degree (PAD) to determine whether health resources are allocated fairly in different areas within a region [51]. HRAD and PAD were evaluations of GP resource allocation based on geographic and demographic perspectives. Respectively, the ratio of HRAD/PAD can show whether local healthcare resources were meeting the needs of local residents. We used HRAD to measure the degree of agglomeration of GP in a specific area and the differences between different areas. HRAD/PAD was used to measure whether regional health resources were meeting the needs of the local population [11].

The formula for agglomeration degree based on geographical area is:

$${\text{HRADi }} = {\text{ HRi}}/{\text{HRn}} \times {1}00\% /{\text{Ai}}/{\text{An}} \times {1}00\% \, = \, \left( {{\text{HRi}}/{\text{HRn}}} \right)/\left( {{\text{Ai}}/{\text{An}}} \right)$$

In this equation, HRADi refers the HRAD of region i, HRi is the number of health resources in region i, HRn is the total health resources of the whole region at the previous level, Ai is the geographic area of region i, An is the total geographic area of the region at the previous level. In this paper, HRi represents the number of GPs in region i, HRn represents the total number of GPs in China. Ai represents the geographic area of region i, An represents the geographic area of China. When HRAD is greater than 1, it indicates that the geographical distribution of GP is fair; when HRAD is less than 1, it indicates that the geographical distribution of GP resources is poor; when HRAD is equal to 1, the geographical distribution of health resources is absolutely fair.

The formula based on population is:

$${\text{PADi}} = {\text{ Pi}}/{\text{Pn}} \times {1}00\% /{\text{Ai}}/{\text{An}} \times {1}00\% = \left( {{\text{Pi}}/{\text{Pn}}} \right)/\left( {{\text{Ai}}/{\text{An}}} \right)$$

In this paper, where PADi denotes the degree of population agglomeration in region i. Pi represents the number of population in region i, Pn represents the total population of China, Ai denotes the geographic area of region i, An denotes the total geographic area.

When HRAD/PAD is greater than 1, it means that the GP resources in the region are sufficient; when HRAD/PAD is less than 1, it means that the GP resources in the region are relatively scarce; when HRAD/PAD is close to 1, it means that the GP resources in the region can guarantee the needs of local residents [51].

### GM (1,1) model

The Grey prediction model generates a regular sequence by cumulative calculation of the original data to predict future trends [52, 53]. This method has low requirement on original data, high accuracy, adaptability, was favored by scholars [54, 55]. The univariate Grey prediction model was mostly used to reveal its internal development law through the univariate first-order differential equation, which is mainly used in the single time series forecasting, the most classical one is GM (1,1) model [56, 57]. In the GM (1,1) model, it is necessary to calculate the development coefficient A, the endogenous control gray numbers B, as well as to calculate the posterior variance ratio C and the posterior probability P. The development coefficient A represents the developmental dynamics of the estimated value of the behavioral series; the endogenous control gray numbers B is the data mined from the behavioral series and reflects the relationship of the data changes. When the data is singly increasing or decreasing, the accuracy is above 98%. The steps of establishment and accuracy evaluation metrics of GM (1,1) model were provided in Additional file 1 in detail.

### Data analysis

The SPSS 25.0 software was used to collect data from GPs in mainland China and the data was analyzed descriptively. The HRAD index, the PAD index, the Gini coefficient, the Lorenz curve were calculated using Stata 16.0. The GM (1,1) model was constructed, run and validated using Python 3.0.

## Results

### Basic information of GPs in China

Since the establishment of the GP system in China in 2011 and its formal classification as a specialized type of doctor, the number of GPs in China has increased significantly. In 2021, the total number of GPs on the Chinese mainland was 434 868, about four times that in 2012 (109 794), representing a total increase of 325 074 over the decade, with an annual growth rate of 16.53%. Of these, 314 279 were registered as GPs. After 10 years of development, the number of GPs with GP training certificates and who successfully registered has increased from 33.86% to 72.27%, changing the low registration rate phenomenon, their composition ratio has changed significantly. The percentage of those who obtained the GP training certificate has decreased year after year (the Chinese government has not announced the number of those who obtained the GP training certificate in 2021).

From the perspective of each province, the growth rate of GPs in Guizhou (27.62%), Shaanxi (24.65%), Henan (24.46%) were at the top of the list over the 10 years, except for Tibet, where no comparison was made due to the low base of GPs; Jiangsu, Guangdong and Sichuan provinces saw the largest increase in the number of GPs on the Chinese mainland, with 34 560, 29 237 and 20 548 GPs added respectively over the past 10 years. Regionally, the number of GPs in 2021 was 434 868, including 224 229 in the Eastern region, 113 757 in the Central region, 96 882 in the Western region, representing 51.56%, 26.16%, 22.28%, respectively. The 10-year average growth rates for GPs in the Eastern region, Central, Western region were 14.48%, 19.91%, 18.39%, respectively, with 157 828, 91 565, 75 681 new GPs. The Eastern region had the highest rate of growth in the number of GPs and the Central region the highest annual rate. Tables 1 and 2 show the detailed results.

**Table 1:** Current situation of GPs in China (2012–2021)

Year	Total number of GPs	Number of registered GPs (n (%))	The number of people who have obtained training certificates for GPs (n (%))	Number of GPs per 10 000 population
2012	109 794	37 173 (33.86%)	72 621 (66.14%)	0.81
2013	145 511	47 402 (32.58%)	98 109 (67.42%)	1.07
2014	172 597	64 156 (37.17%)	108 441 (62.83%)	1.27
2015	188 649	68 364 (36.24%)	120 285 (63.76%)	1.37
2016	209 083	77 631 (37.13%)	131 452 (62.87%)	1.51
2017	252 717	96 235 (38.08%)	156 482 (61.92%)	1.82
2018	308 740	156 800 (50.79%)	151 940 (49.21%)	2.22
2019	365 082	210 622 (57.69%)	154 460 (42.31%)	2.61
2020	408 820	255 867 (62.59%)	152 953 (37.41%)	2.90
2021	434 868	314 279 (72.27%)	–	3.08

–, that the Chinese government has not published this item of data

**Table 2:** The Number of GPs in different regions of China (2012–2021)

Area	2012	2013	2014	2015	2016	2017	2018	2019	2020	2021	Average growth rate (%)
Total	109 794	145 511	172 597	188 649	209 083	252 717	308 740	365 082	408 820	434 868	16.53
Eastern region	66 401	84 464	96 979	104 015	116 537	139 473	170 362	192 116	207 862	224 229	14.48
Beijing	8137	8458	8221	8269	8402	8591	8861	9267	9918	9303	1.50
Tianjin	1095	1427	1622	2144	2403	3749	4138	4568	5051	5615	19.92
Hebei	3493	6730	8637	9286	9355	10 017	11 292	18 407	18 995	24 410	24.11
Liaoning	3304	3513	3777	3624	4195	6273	9002	10 847	11 771	11 922	15.32
Shanghai	5323	5957	6925	7352	7967	8491	8629	9924	9876	10 673	8.04
Jiangsu	15 068	17 650	19 748	20 841	25 162	27 578	47 794	47 601	49 628	49 433	14.11
Zhejiang	12 251	17 041	19 640	21 627	22 571	30 467	26 047	27 406	27 628	23 446	7.48
Fujian	2594	3634	4310	5122	5786	6897	8182	9157	10 145	11 644	18.16
Shandong	6775	7709	8967	9920	11 372	13 565	17 426	21 034	24 760	35 914	20.36
Guangdong	7940	11 765	14 404	14 955	18 338	22 712	27 638	31 950	37 177	39 016	19.35
Hainan	421	580	728	875	986	1133	1353	1955	2913	2853	23.69
Middle region	22 192	29 674	39 020	45 344	49 944	63 269	75 302	94 847	106 306	113 757	19.91
Shanxi	2552	2958	3618	4014	4175	6372	5962	6516	7033	7441	12.63
Jilin	1231	1680	2299	2891	3384	5130	4965	7536	7992	8272	23.57
Heilongjiang	2081	2889	3730	4320	4454	4493	5637	6593	6942	6906	14.26
Anhui	3191	4319	6814	7360	8625	10 430	12 917	15 116	18 501	17,101	20.51
Jiangxi	2081	2429	3020	3319	3641	5268	5620	6705	8031	9624	18.55
Henan	4722	6427	8394	10 349	12 129	15 567	20 497	22 763	24 358	33 830	24.46
Hubei	3752	5044	6090	6970	7020	8969	10 863	12 857	13 847	12 625	14.43
Hunan	2582	3928	5055	6121	6516	7040	8841	16 761	19 602	17 958	24.05
Western region	21 201	31 373	36 598	39 290	42 602	49 975	63 076	78 119	94 652	96 882	18.39
Inner Mongolia	1679	2374	2937	3085	3178	3986	4894	5801	6042	6103	15.42
Guangxi	3087	4039	4527	4671	5104	6275	7958	10 662	13 149	13 091	17.41
Chongqing	1632	2187	2527	2872	3127	3866	6348	8117	8769	8944	20.81
Sichuan	4665	8983	9819	10 394	10 360	11 343	13 404	17 838	25 213	20 776	18.05
Guizhou	1032	1511	2416	3147	3714	5014	6238	6466	7572	9269	27.62
Yunnan	3212	4261	4106	4289	4737	5253	6381	8812	9481	9250	12.47
Tibet	34	67	109	161	202	247	352	642	730	467	33.79
Shaanxi	1824	1978	2770	2126	2738	3578	4979	5300	8098	13 255	24.65
Gansu	1389	2106	2710	3312	3773	3824	4835	5994	6516	7422	20.47
Qinghai	462	758	881	961	993	1230	1315	1514	1625	1686	15.47
Ningxia	260	392	471	565	654	926	1279	1500	1638	1627	22.60
Xinjiang	1925	2717	3325	3707	4022	4433	5093	5473	5819	4992	11.17

In 2021, GPs have grown to 10.14% of all practicing (assistant) physicians. According to the results of the geographical division, with the highest percentage of 11.54% in the Eastern region, 9.17% and 8.78% in the Central and Western regions. From a national perspective, the number of GPs per 10 000 people will be 3.08 by 2021, with a regional breakdown showing a high of 3.69 per 10 000 in the Eastern region and a slight difference of 2.71 per 10 000 in the Central region and 2.53 per 10 000 in the Western region. By 2021, the number of GPs per 10 000 population in mainland China has gradually increased from 0.81 in 2012 to 3.08 in 2021, China has successfully achieved the goal set when the GP system was launched in 2012, to initially establish a GP system by 2020, basically achieve 2–3 qualified GPs per 10 000 residents in urban and rural areas, initially meet the medical needs of the residents. From an overall perspective, the ratio of GPs in the three regions was still at a low level. See Table 3 for details.

**Table 3:** Number of GPs in three regions of China in 2021

Area	Total number of GPs	Number of GPs per 10 000 population
Total	434 868	3.08
Eastern region	224 229	3.69
Middle region	113 757	2.71
Western region	96 882	2.53

### Analysis of GP allocation

#### Lorenz curve and Gini coefficient

**Fig. 1:**
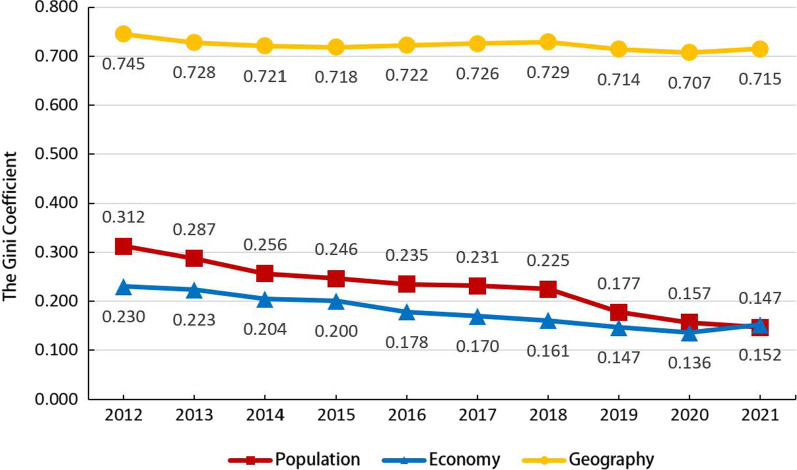
Trend of Gini coefficient in population/economic/geographical dimensions of Chinese GPs in 2012–2021

From 2012 to 2021, the Gini coefficient of the distribution of GPs by population in China decreased from 0.312 to 0.147, the Gini coefficient of the economic perspective decreased from 0.230 to 0.152. In addition, the Gini coefficient of allocation of GPs by geography remained between 0.700 and 0.750. By plotting the trend of Gini coefficient changes in the population/economic/geographic dimensions of Chinese GPs from 2012 to 2021 (Fig. 1), we can see a decreasing trend in the Gini coefficient for all three dimensions, the decreasing trend was more obvious in the demographic and economic dimensions. Which means that the equity level of allocation of Chinese GPs improved faster in the demographic and economic dimensions. While the geographic dimension of the Gini coefficient has a smaller tendency to decrease, it fluctuates from 0.745 to 0.715 over a decade. The Gini coefficients of the demographic and economic dimensions were both below the 0.2 (below the warning line of 0.40), indicating that their GP allocation levels were more equitable and reasonable in the demographic and economic dimensions. However, in the geographic dimension, the Gini coefficients were far above the warning line (above 0.7), indicating that in the geographic dimension, the disparity of GP resource allocation in China was large.

In a Lorenz curve, the diagonal from the origin of the coordinate to the vertex in the upper right corner of the square was called the line of absolute equality. As can be seen from the Lorenz curves of GP allocation from 2012 to 2021, the radians of the Lorenz curves of the population dimension and the economic dimension become smaller, the residual region between them and the absolute line of equity becomes smaller. The Lorenz curve of the population dimension gradually approaches the Lorenz curve of the economic dimension over the course of a decade, both reach a fairer level. This indicates a more equitable allocation of resources. In contrast, the radian of the geographical dimension was the largest, with a slight change over the past ten years, which was significantly different from the Lorenz curve of the population and economic dimensions, further away from the absolute fair line, indicating that the equity of GP resources allocation in the geographical dimension was poor in mainland China. Detailed results were shown in Additional file 2: Fig. S1.

#### Agglomeration degree

From the regional classification analysis, it was found that the agglomeration degree in the Eastern region was 4.618 in 2021, which was much higher than 1, indicating that the concentration of GP assignments was far too high; the Central region has an agglomeration degree of 1.493, which was slightly greater than that of, indicating that the distribution of GPs was relatively fair; The agglomeration degree of GP in the Western region was 0.312, which is much lower than 1, this level was highly unfair to the distribution of GPs. Analyzing the agglomeration degree of each province, autonomous region and municipality directly under the central government, the agglomeration degree of Guangxi (1.221), Guizhou (1.166), Shanxi (1.052), Jilin (0.978), Sichuan (0.947) were around 1, which tends to be absolutely fair. Some regions have an agglomeration degree less than one. Heilongjiang (0.324), Inner Mongolia (0.114), Xinjiang (0.066), Qinghai (0.052), Tibet (0.008) have a low agglomeration degree, which indicates a highly inequitable allocation of GPs based on geographic regions. In contrast, Shanghai (37.546), Beijing (12.572), Tianjin (10.370), Jiangsu (10.220) have values of agglomeration degree in these regions that exceed 10, indicating that their GP allocations were too concentrated in these regions. The results for the agglomeration degree of population showed that the agglomeration degree in 2021 was 1.196, 0.880, 0.821 in the Eastern, Central, Western regions, respectively. There was still a concentration of GPs in the Eastern region. The distribution levels of GPs in the Central and Western regions were similar, the resources for GP were relatively scarce. From the perspective of provinces, autonomous regions, municipalities directly under the central government, based on population agglomeration degree, most regions were between 0.8 and 1.2, indicating that the overall distribution of GPs was more balanced in terms of population distribution, however, GP resources in Jiangsu (1.885) were still concentrated.

From 2012 to 2021, the agglomeration degree of GP in the Eastern region decreased from 5.416 to 4.618, in the Central region increased from 1.153 to 1.493, in the Western region increased from 0.271 to 0.312. Analyzing the agglomeration degree of each region, Shanghai, Jiangsu, Beijing and Zhejiang all had an agglomeration degree above 10 in 2012, especially Shanghai and Beijing, which reached 43.553 and 74.167, respectively, after a decade of development, the agglomeration degree of each region decreased. In Beijing, for example, the agglomeration degree of GPs fell from 43.553 in 2012, at an annual rate of − 12.90%, to 12.572 in 2021, while in Shanghai, it fell from 74.167 in 2012, at an annual rate of − 7.28%, to 37.546 in 2021. The GP in Liaoning, Inner Mongolia, Qinghai, Guangxi showed a relatively stable situation (− 1% to 1%) in terms of population agglomeration degree in the past ten years. In contrast, the four provinces of Henan, Hebei, Hunan, Hainan were still growing with a population agglomeration degree of 1 in 2012, the equity of their GP resources in terms of PAD is becoming more and more unequal. Detailed results were shown in Additional file 2: Table S3.

### GM (1,1) model

**Fig. 2:**
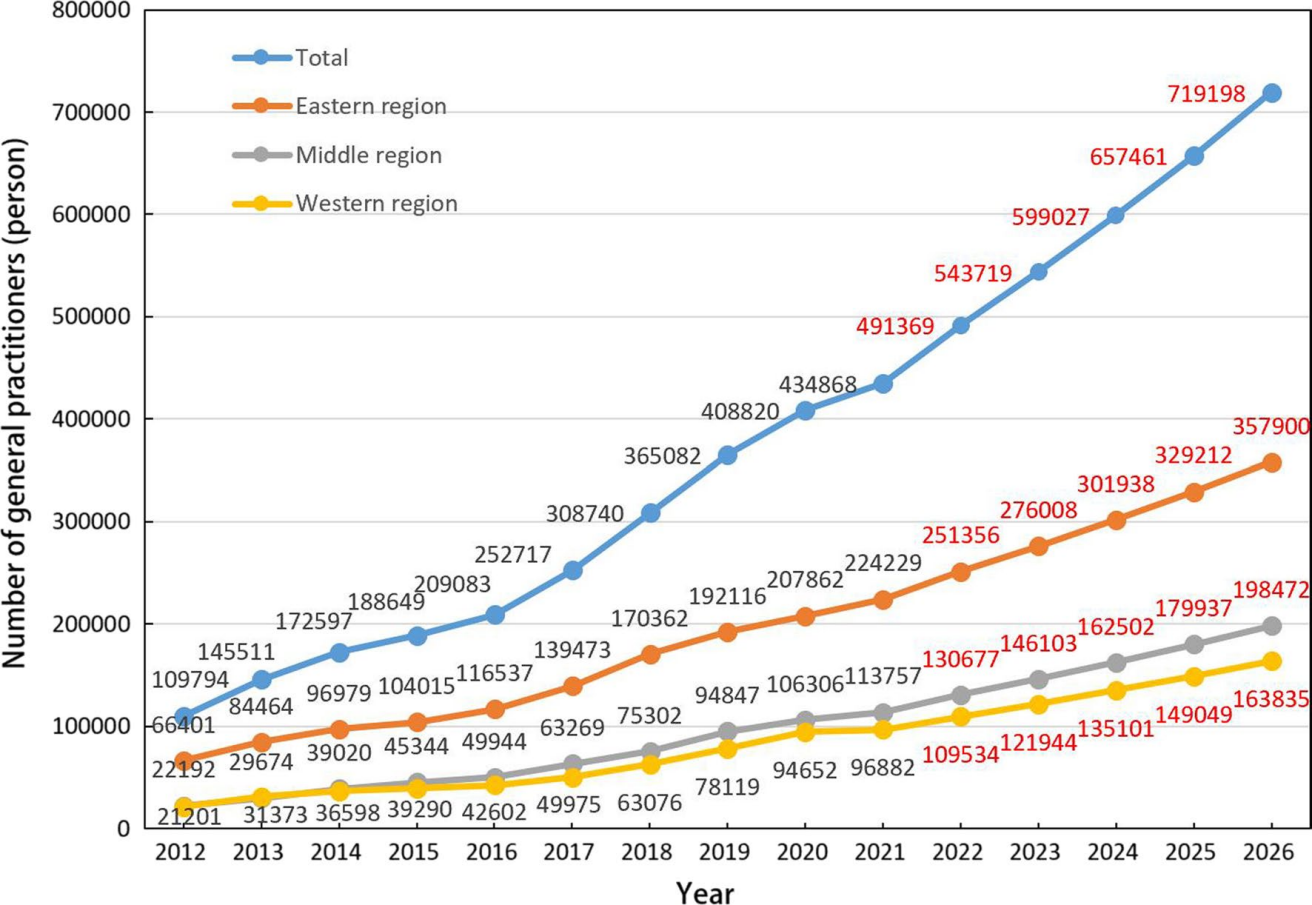
Growth and forecast of GPs in China (2012–2026)

In this study, the GM (1,1) model was established using the original series of GP resources data in mainland China from 2012 to 2021, the number of GPs was assigned to the time series for a total of 10 years from 2012 to 2021. The number of GPs in mainland China in the next 5 years (2022–2026) was obtained through the calculation formula. The prediction results of the number of GPs in the whole country and the Eastern, Central and Western regions were modeled from the regional perspective, the model prediction results were tested by the posterior deviation test, the test statistic *C* was less than 0.35 and the P-value was greater than 0.95, which indicated that the model accuracy level was very good and the model was well adapted, the accuracy level of each model is 1. Therefore, the model can be applied to extrapolating prediction studies of the number of GPs and the population of mainland China. The results showed that the development coefficients *A*, which were − 0.055, − 0.051, − 0.061 and − 0.058 for the whole country, Eastern, Central and Western regions respectively. All values were less than 0.3, indicating that they can be used for medium- and long-term forecasting with high accuracy. The endogenous control gray numbers *B* were 519 387, 279 536, 128 328, 111 627, respectively (Table 4). The predicted number of GPs in 2026 calculated by the GM (1,1) model based on the original data were 719 198 nationwide, 357 900 in the East, 198 472 in the Central, 163 835 in the West (all within 5% error at the later stage), as shown in Table 5. After a decade of development since the establishment of the GP system, the GPs in the Chinese mainland in 2021 showed a continuous growth trend. The number of GPs in mainland China has reached 430 000, an increase of approximately 320 000 or 396% compared to 109 794 in 2012. By forecasting the next 5 years, it is expected that the number of GPs in mainland China will reach 720 000 in 2026, an increase of about 280 000 (65.38%) compared to 2021. The Eastern region, the Central region and the Western region grew by about 133 671, 84 715, 66 953 respectively, an increase of 59.61%, 74.47% and 69.11%. See Fig. 2 for details.

**Table 4:** GM (1,1) model test value

Project	East region	Central region	West region	Total
Development coefficients A	− 0.051	− 0.061	− 0.058	− 0.055
Endogenous control gray numbers B	279 536	128 328	111 627	519 387
Test statistic C	0.013	0.011	0.035	0.014
P-value	1	1	1	1

**Table 5:** Number of GPs by region predicted by GM (1,1) model (2022–2026)

Year	East region	Central region	West region	Total
2022	251 356	130 677	109 534	491 369
2023	276 008	146 103	121 944	543 719
2024	301 938	162 502	135 101	599 027
2025	329 213	179 937	149 049	657 461
2026	357 900	198 472	163 835	719 198

In addition, this study also predicted the population size of mainland China in the next 5 years and used it to calculate the number of GPs per 10 000 population in 2026, which showed that the population size of mainland China will reach 1.451 billion in 2026 and the number of GPs per 10 000 population will reach 4.9 [58]. The number of people in the Eastern, Central and Western regions will reach 642, 415 and 394 million, respectively. The number of GPs per 10 000 population will reach 5.5, 4.8, 4.1, which is basically consistent with the government’s proposal in 2018 to reach 700 000 GP by 2030, reaching the goal of 5 GPs per 10 000 people. However, the actual number of GPs in mainland China may be lower than the results of the predicted model, also the number of GPs per 10 000 population may be lower than the results shown in the study, due to factors such as the impact of the COVID-19 epidemic, saturation of GP resources or job restrictions in high agglomeration degree areas. Table 6.

**Table 6:** Population forecast and number of GPs per 10 000 people in Mainland China

Year	East region		Central region		West region		Total	
	Predictive value	GPs/10 000 people	Predictive value	GPs/10 000 people	Predictive value	GPs/10 000 people	Predictive value	GPs/10 000 people
2022	62 186	4.0	41 849	3.1	38 772	2.8	142 773	3.4
2023	62 866	4.4	41 734	3.5	39 005	3.1	143 546	3.8
2024	63 554	4.8	41 619	3.9	39 239	3.4	144 324	4.2
2025	64 249	5.1	41 505	4.3	39 474	3.8	145 106	4.5
2026	64 952	5.5	41 391	4.8	39 711	4.1	145 893	4.9

## Discussion

This is a longitudinal study that assessed changes in GP in mainland China from 2012 to 2021. The total number of GPs in China has shown steady growth, with a significant increase in the total number of GPs and the number of GPs per 10 000 people, as well as an increase in the number of people registered as GPs. The number of GPs in China has reached 430 000, with the number of GPs per 10 000 people gradually increasing from 0.81 per 10 000 in 2012 to 3.08 per 10 000 in 2021. When the GP system was set up in 2011, it was proposed that there should be 2–3 qualified GPs for every 10 000 urban and rural residents by 2020. The Chinese GP team has a positive development trend and has mostly achieved relevant policy goals. However, due to China’s vast territory, geographic inequity remains an unavoidable problem in resource allocation. Not only China but also other developing countries, such as Mexico and Vietnam, have serious inequalities in the geographical distribution of health workers [59]. Regardless of the number of GP per 10 000 people or the number of GP per 100 square kilometers, the number of GP allocation in economically developed coastal areas such as the Eastern region is much more than that in economically underdeveloped areas such as the Western region. The Lorenz curve of GP distribution in terms of service population and gross domestic product in mainland China is close to the fair line, the Lorenz curve in terms of population dimension has gradually approximated the Lorenz curve in terms of economic dimension over the past decade to reach the fair level. However, the Lorenz curve distributed by geographical area is far from the fair line, which is obviously different from Lorenz curve of population and economic dimension. From the results of Gini coefficient, after ten years of development, the GPs in mainland China have reached a relatively fair level in both the population dimension and the economic dimension. The geographical distribution of the Gini coefficient is less volatile. Three dimensions of the Gini coefficient are declining, the population dimension and economic dimension downward trend are more obvious. With the help of the central government and local governments, the equity of GP allocation has been greatly improved in the demographic and economic dimensions, while in the geographical dimension, the Gini coefficient does not fluctuate much, and it also shows a slow downward trend.

According to the results of GP agglomeration degree, provinces with an average population distribution and sparsely populated provinces have low GP agglomeration, the Western region of 2021 has a GP agglomeration degree much lower than 1. This level of GP allocation is not fair. However, the agglomeration degree of GP is higher in densely populated provinces such as Shanghai, Jiangsu, Beijing, Zhejiang. In a few regions, such as Liaoning and Inner Mongolia, the fluctuation of GP resources in population agglomeration degree in the past decade is small. Compared to 2012, the agglomeration degree of GP and population agglomeration degree have changed to varying degrees. The Eastern region, which has a high degree of agglomeration, has achieved some results due to the efforts of the provinces, while some regions with a high degree of agglomeration have decreased to varying degrees. The agglomeration degree in the West has improved somewhat, however, there is still a lot of room for improvement. The reasons are as follows: on the one hand, the tilt of the national policy and the increase of financial input enable the Western provinces (municipalities directly under the central government and autonomous regions) to invest more resources in the development of medical services, thus increasing the total amount of health resources. In recent years, in order to accelerate the construction of GP team and strengthen the training of GPs, China has issued a series of relevant policies, which have promoted the development of GP to a large extent and achieved certain results. Therefore, when making policies, the government should give full consideration to the level of economic development and reduce the unfairness caused by economic factors. While promoting the development of GP teams in the Central and Western regions, we encourage the developed Eastern regions to improve the quantity and quality of GP resources and optimize the GP team structure by taking advantage of their own economic advantages. Each region should focus on reducing differences within the region, focus on the overall development of its own economy, take measures based on local conditions and in line with its own situation, improve relevant policies for GP in the region. Maintain the positive trend of GP development, give full play to the role of GP health “gatekeeper [23]”.

The development of GP in China started late, there is a large gap between the level of GP allocation and foreign countries [60]. Major developed countries and some developing countries have established a relatively complete GP system. In the United States, Britain, Canada and Australia, there is one GP for every 2000–3000 people, its GP team has accounted for nearly half of the total number of doctors, enjoy higher welfare benefits and social status, China is still far from this goal [61]. In addition, the workload of Chinese GP is heavy, accompanied by excessive pressure and low income [62]. With the increasing aging problem in China, the work of Chinese general practitioners has become increasingly stressful [63]. Due to an increase in patients and a shortage of medical staff in the wake of the COVID-19 pandemic, the workload of Chinese GP is becoming more and more heavy. A survey has found that 35.20% of GPs have a strong intention to quit, low material satisfaction and low growth satisfaction are the main influencing factors of GP dismission [64, 65]. The low income and low social status of the GP community are also major barriers to medical graduates becoming GPs [11, 48]. Although there are policies requiring “establishing incentive mechanism for GP” and “broadening the career development path of GP”, they are only principled initiatives without rigid binding force, local implementation initiatives are not strong [23]. Therefore, this must be urgently rectified through financial incentives. The Healthy China 2030 Health System Reform Plan and a recent directive from the Chinese government to prioritize primary health care and GP training by strengthening pay incentives for trainees are an essential step forward [66]. Additionally, given China’s 1.3 billion people and 9.6 million square kilometers, the past investments have been far from adequate. More incentive policies are needed to motivate the recruitment and retention of GP in the Central and Western regions, including the transformation of medical graduates to GP, the improvement of GP registration rate, the salary of GP, professional promotion of GP, the living conditions of GP and their families, additional compensation.

From 2022 to 2026, based on natural growth trends, the total number of GPs in China will be about 720 000, the number of GPs per 10 000 population will reach 5.5, 4.8, 4.1 in the Eastern, Central, Western region, respectively. According to the GM (1,1) model. The population of mainland China will reach 1.451 billion and the number of GPs per 10 000 population will reach 4.9. The development trend of GP resources in China is positive, which is fundamentally similar to the target set, the target may be reached ahead of schedule [67].

## Conclusion

China has invested heavily in the GP system over the past decade and introduced various policies to promote its development. Therefore, it is necessary to analyze equity in GP distribution to ensure equal access to health resources for all residents. Our study shows that after a decade of development, the number of GPs in China has increased significantly. The goal of the GP system when it was set up has been achieved. However, there are still some shortcomings. GPs are unevenly distributed across regions, with large regional variations. There is a shortage of GPs in the Western region, both in terms of population size and geographical area, while resources are over-concentrated in the Eastern region. In the future, GPs in mainland China will continue to maintain a rapid growth trend, supported by policies. The actual situation may differ considerably from the predicted results, overall, the development of GPs in China will enter a period of rapid development. Therefore, the government should adhere to the policy guidance and continuously increase financial input to support the development of GPs in Western regions, scientifically and reasonably control the oversupply of GPs in Eastern and Central regions, promote the healthy and sustainable development of GPs in China.

## Research limitations

Firstly, this study evaluates the equity of GP allocation based on the assumption of resource homogeneity. The study did not distinguish between differences in service quality and capacity among GPs. Second, this study assesses the equity of GP allocation based on population, economy, geography from a provincial perspective, which may not be highly accurate and precise. However, the higher the level of economic development in a region, the higher the level of education and living standards of its residents, therefore the corresponding increase in the demand for human resources for health. The interplay between these is not discussed. The final prediction of the GP is a trend of growth in a state of nature independent of external influences, which does not take into account the effects of many actual factors. In the future, we will take into account more influencing factors, using more accurate and reasonable methods to assess and analyze China's GPs.

## Supplementary Information

**Additional file 1: Table S1.** Number of GPs in China from 2012 to 2021. **Table S2. **Model prediction accuracy test. **Table S3. **Model test results. **Table S4.** The Posterior Deviation criterion of predictive accuracy for the GM (1,1).

**Additional file 2: Figure S1. **Lorenz curve of Chinese GPs in 2012–2021. **Table S5.** Agglomeration degree in 2012–2021.

## Data Availability

The data used in this paper are from China Statistical Yearbook and China Health and Family Planning Yearbook.
