# Supplementary material for: Equity and trends in general practitioners’ allocation in China: based on ten years of data from 2012 to 2021
Source: Hum Resour Health. 2023 Aug 2;21:61. doi: 10.1186/s12960-023-00841-5 (PMC10394803; doi:10.1186/s12960-023-00841-5)
Supplement: Supplementary file 1 — Additional file 1: Table S1. Number of GPs in China from 2012 to 2021. Table S2. Model prediction accuracy test. Table S3. Model test results. Table S4. The Posterior Deviation criterion of predictive accuracy for the GM (1,1). [file 12960_2023_841_MOESM1_ESM.docx]

**Additional file 1: Methods S1**

**Table S1** Number of GPs in China from 2012 to 2021.

| **Years** | **East** | **Central** | **West** | **Total** |
| --- | --- | --- | --- | --- |
| 2012 | 66401 | 22192 | 21201 | 109794 |
| 2013 | 84464 | 29674 | 31373 | 145511 |
| 2014 | 96979 | 39020 | 36598 | 172597 |
| 2015 | 104015 | 45344 | 39290 | 188649 |
| 2016 | 116537 | 49944 | 42602 | 209083 |
| 2017 | 139473 | 63269 | 49975 | 252717 |
| 2018 | 170362 | 75302 | 63076 | 308740 |
| 2019 | 192116 | 94847 | 78119 | 365082 |
| 2020 | 207862 | 106306 | 94652 | 408820 |
| 2021 | 224229 | 113757 | 96882 | 434868 |

**The establishment of GM (1,1) model**

The steps of establishment of GM (1,1) model are as follows:

Step 1. Suppose the original data sequence $X^{(0)}$with *n* sample showed in Eq.(1).

$X^{(0)}=\left( x^{\left( 0 \right)}\left( 1 \right),x^{\left( 0 \right)}\left( 2 \right){,x}^{\left( 0 \right)}\left( 3 \right),\cdots{,x}^{\left( 0 \right)}(n) \right) n\geq4$ (1)

Where $x^{\left( 0 \right)}(i)$>0, *i*=1,2,3⋅⋅⋅*n*. Then the $X^{\left( 0 \right)}$is transformed to the monotonically increasing series $X^{\left( 1 \right)}$using a first time AGO (1-AGO) showed in Eq.(2). This aims to weaken the random factors of the original time series data and strengthen its regularity. After that, we establish the differential equation of the generated number from which the data can be predicted and estimated.

$X^{(1)}=\left( x^{\left( 1 \right)}\left( 1 \right),x^{\left( 1 \right)}\left( 2 \right){,x}^{\left( 1 \right)}\left( 3 \right),\cdots{,x}^{\left( 1 \right)}(n) \right)$ (2)

where,

$x^{\left( 1 \right)}\left( k \right)=\sum_{i=1}^{k} x^{\left( 0 \right)}\left( i \right) k=1,2,3,\cdots,n$ (3)

Step 2. A first-order grey differential equation is formed to obtain GM (1,1) model shown in Eq. (4).

$x^{\left( 0 \right)}\left( k \right)+aZ^{\left( 1 \right)}(k)=b k=2,3,4,\cdots,n$ (4)

where,

$Z^{\left( 1 \right)}(k)=\frac{1}{2}[x^{\left( 1 \right)}\left( k \right)+x^{\left( 1 \right)}\left( k-1 \right)] k=2,3,4,\cdots,n$ (5)

Where ‘a’ represent the developing coefficient and ‘b’ represent the driving coefficient. $Z^{\left( 1 \right)}(k)$is the background value of $x^{\left( 0 \right)}(k)$.The two parameters ‘a’ and ‘b’ can be estimated by the least square method $\left[ a,b \right]^{T}$shown in Eq.(6).

$\left[ \begin{matrix} a \\ b \end{matrix} \right]={[\begin{matrix} B^{T} & B \end{matrix}]}^{-1}B^{T}Y$ (6)

Where ‘Y’ is the constant vector, and ‘B’ is the accumulated matrix showed in Eq.(7) and Eq.(8).

$Y=\left[ \begin{matrix} x^{\left( 0 \right)}(2) \\ \begin{matrix} x^{\left( 0 \right)}(3) \\ x^{\left( 0 \right)}(4) \\ \vdots\end{matrix} \\ \begin{matrix} \vdots\\ x^{\left( 0 \right)}(n) \end{matrix} \end{matrix} \right]$ (7)

$B=\left[ \begin{matrix} {-Z}^{\left( 1 \right)}(2) & 1 \\ {-Z}^{\left( 1 \right)}(3) & 1 \\ \begin{matrix} {-Z}^{\left( 1 \right)}(4) \\ \begin{matrix} \vdots\\ \vdots\\ {-Z}^{\left( 1 \right)}(n) \end{matrix} \end{matrix} & \begin{matrix} 1 \\ \begin{matrix} \vdots\\ \vdots\\ 1 \end{matrix} \end{matrix} \end{matrix} \right]$ (8)

Step 3. The approximate differential equation (whitening equation) model is established by using the discrete data sequence as Eq.(9).

$\frac{{dX}^{(1)}}{dk}+aX^{\left( 1 \right)}=b$ (9)

The predicted value,$\hat{x}^{\left( 1 \right)}(k+1)$, can be obtained by solving the differential equation with initial condition $x^{\left( 1 \right)}\left( 1 \right)=x^{\left( 0 \right)}\left( 1 \right)$.

$\hat{x}^{\left( 1 \right)}(k+1)=\left[ x^{\left( 0 \right)}\left( 1 \right)-\frac{b}{a} \right]e^{-ak}+\frac{b}{a} k=1,2,3,\cdots,n$ (10)

Finally, by means of the IAGO, the predicted value, $\hat{x}^{\left( 0 \right)}(k+1)$, can be calculated as follows:

$\hat{x}^{\left( 0 \right)}\left( k+1 \right)=\hat{x}^{\left( 1 \right)}\left( k+1 \right)-\hat{x}^{\left( 1 \right)}(k) k=1,2,3,\cdots,n$ (11)

therefore,

$\hat{x}^{\left( 0 \right)}\left( k+1 \right)=(1-e^{a})\left( x^{\left( 0 \right)}\left( 1 \right)-\frac{b}{a} \right)e^{-ak} k=1,2,3,\cdots,n$ (12)

Note that $\hat{x}^{\left( 1 \right)}\left( 1 \right)$=$\hat{x}^{\left( 0 \right)}\left( 1 \right)$holds.

**The accuracy evaluation metrics of GM (1, 1) Model**

Prediction accuracy is an important criterion for evaluating the performance of a forecasting model. In this study, the posterior deviation test was used to estimate the accuracy of the GM (1, 1) model [1-2].

*The Posterior Deviation Test.* It is tested according to the probability distribution of residual. The posterior variance ratio *C* and the posterior probability *P* are two main indicators used to evaluate the accuracy of the model.

In this study, we use GP data from 2012-2021 to make projections from an overall national perspective and from the perspective of each of the three major divisions. To test the prediction accuracy of this model, the data from 2012-2018 were defined as the training dataset and the years 2019-2021 were defined as the test dataset, and based on these two datasets, the predicted values from the national perspective for 2019-2021 are compared with the actual values to check the performance of the prediction model. The results show that all indicators of the tested models meet the criteria, and the model prediction compliance are 90.68% (2019), 90.66% (2020) and 94.89% (2021). The details of the test results are shown in Tables S2 and S3.

**Table S2** Model prediction accuracy test

| Year | Actual value | Predicted value | Degree of conformity |
| --- | --- | --- | --- |
| 2019 | 365082 | 331058.680 | 90.68% |
| 2020 | 408820 | 370621.473 | 90.66% |
| 2021 | 434868 | 412630.682 | 94.89% |

**Table S3** Model test results

| Development coefficients A | Endogenous control gray numbers B | Test statistic C | P-value |
| --- | --- | --- | --- |
| -0.0600 | 408003.7927 | 0.0257 | 1.000 |

Calculate the a posteriori variance ratio *C* as Eq.(13).

$C=\frac{S_{2}}{S_{1}}$ (13)

where,

$S_{1}=\sqrt{\frac{1}{n}\sum_{k=1}^{n} \left[ x^{\left( 0 \right)}\left( k \right)-\bar{x} \right]^{2}}$ (14)

and,

$S_{2}=\sqrt{\frac{1}{n}\sum_{k=1}^{n} \left[ \varepsilon\left( k \right)-\bar{\varepsilon} \right]^{2}}$ (15)

Calculate the posterior probability *P* as Eq.(16).

$P=P\left\{ \left| \varepsilon\left( k \right)-\bar{\varepsilon} \right|<0.6745S_{1} \right\}$ (16)

where,

$\bar{x}=\frac{1}{n}\sum_{k=1}^{n} x^{\left( 0 \right)}(k)$ (17)

$\bar{\varepsilon}=\frac{1}{n}\sum_{k=1}^{n} \varepsilon(k)$ (18)

$\varepsilon\left( k \right)=x\left( k \right)-\hat{x}(k)$ (19)

The smaller the *C* value, the more concentrated the difference between the model predicted value and the actual value. Meanwhile, the larger the *P* value, the less difference between the residual and the residual mean, and the higher fitting accuracy of the GM (1,1) model. According to the size of the value of *C* and *P*, the predictive accuracy rating of the established GM (1,1) model can be graded into four levels “Superior, Qualified, Marginal and Disqualified” [3], as shown in Table S2.

**Table S4** The Posterior Deviation criterion of predictive accuracy for the GM (1,1)

| **Predictive accuracy class** | **posterior variance ratio (C)** | **posterior probability (P)** |
| --- | --- | --- |
| Superior | ≤0.35 | ≥0.95 |
| Qualified | 0.35<C≤0.50 | 0.80≤P<0.95 |
| Marginal | 0.50<C≤0.65 | 0.70≤P<0.80 |
| Disqualified | >0.65 | <0.70 |

References:

1. Wang Y, Wei F, Sun C, et al. The research of improved grey GM (1, 1) model to predict the postprandial glucose in type 2 diabetes[J]. BioMed research international, 2016, 2016.
2. Luo X, Duan H, Xu K. A novel grey model based on traditional Richards model and its application in COVID-19[J]. Chaos, Solitons & Fractals, 2021, 142: 110480.
3. Ye X, Liu J, Yi Z. Trends in the epidemiology of sexually transmitted disease, acquired immune deficiency syndrome (AIDS), gonorrhea, and syphilis, in the 31 provinces of mainland China[J]. Medical science monitor: international medical journal of experimental and clinical research, 2019, 25: 5657.
