# Supplementary material for: Equity and trends in general practitioners’ allocation in China: based on ten years of data from 2012 to 2021
Source: Hum Resour Health. 2023 Aug 2;21:61. doi: 10.1186/s12960-023-00841-5 (PMC10394803; doi:10.1186/s12960-023-00841-5)
Supplement: Supplementary file 2 — Additional file 2: Figure S1. Lorenz curve of Chinese GPs in 2012–2021. Table S5. Agglomeration degree in 2012–2021. [file 12960_2023_841_MOESM2_ESM.docx]

**Additional file 1: Materials**


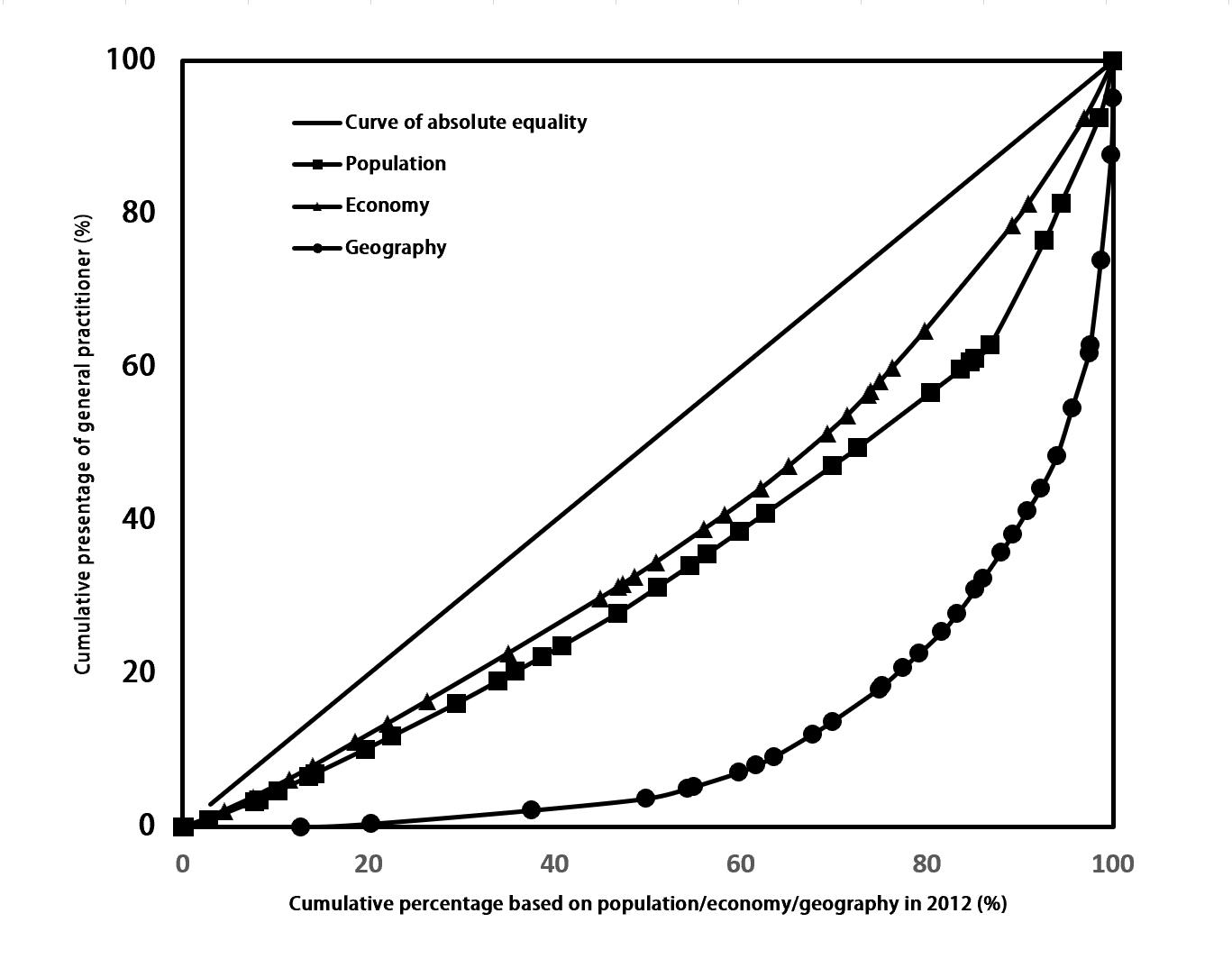


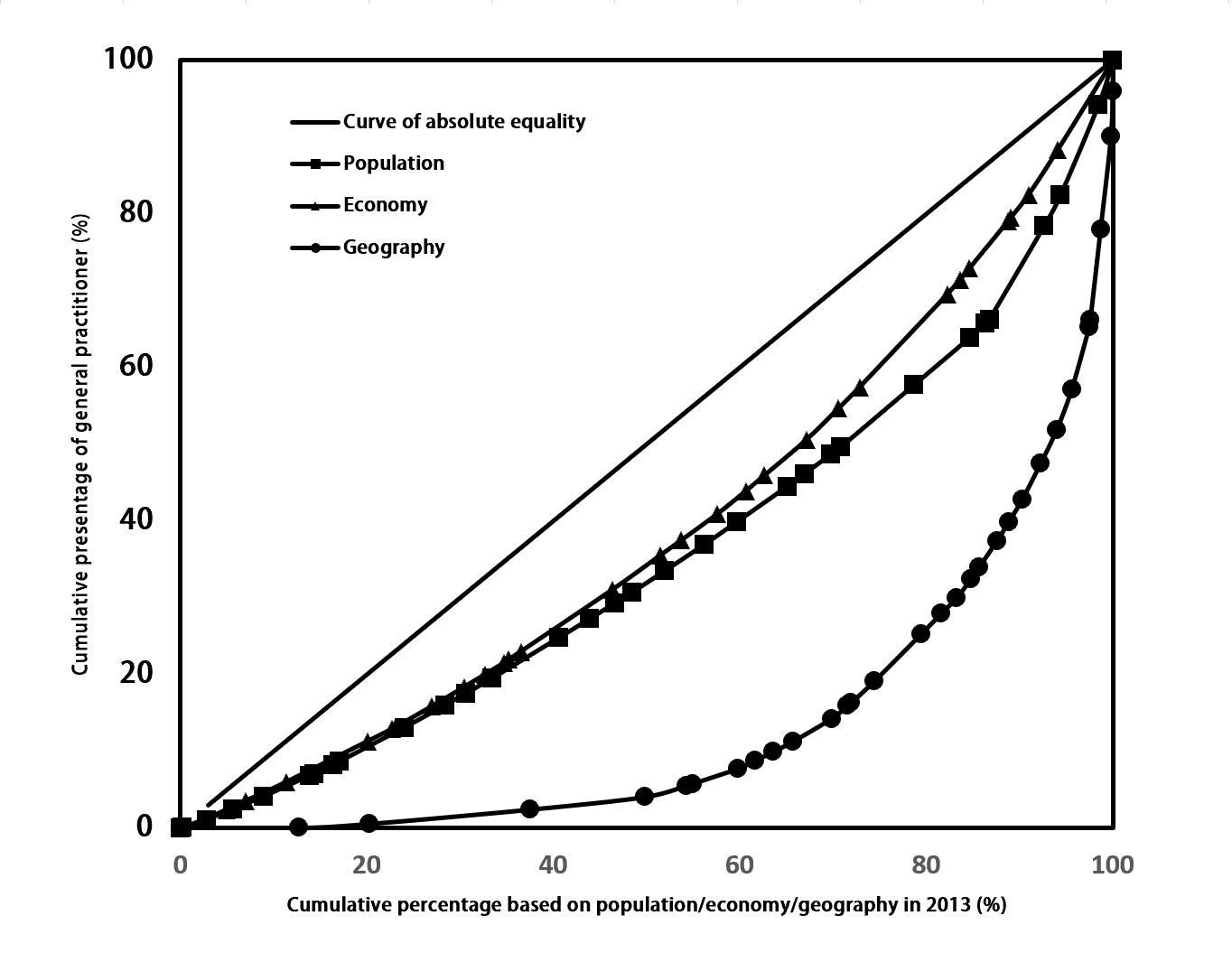


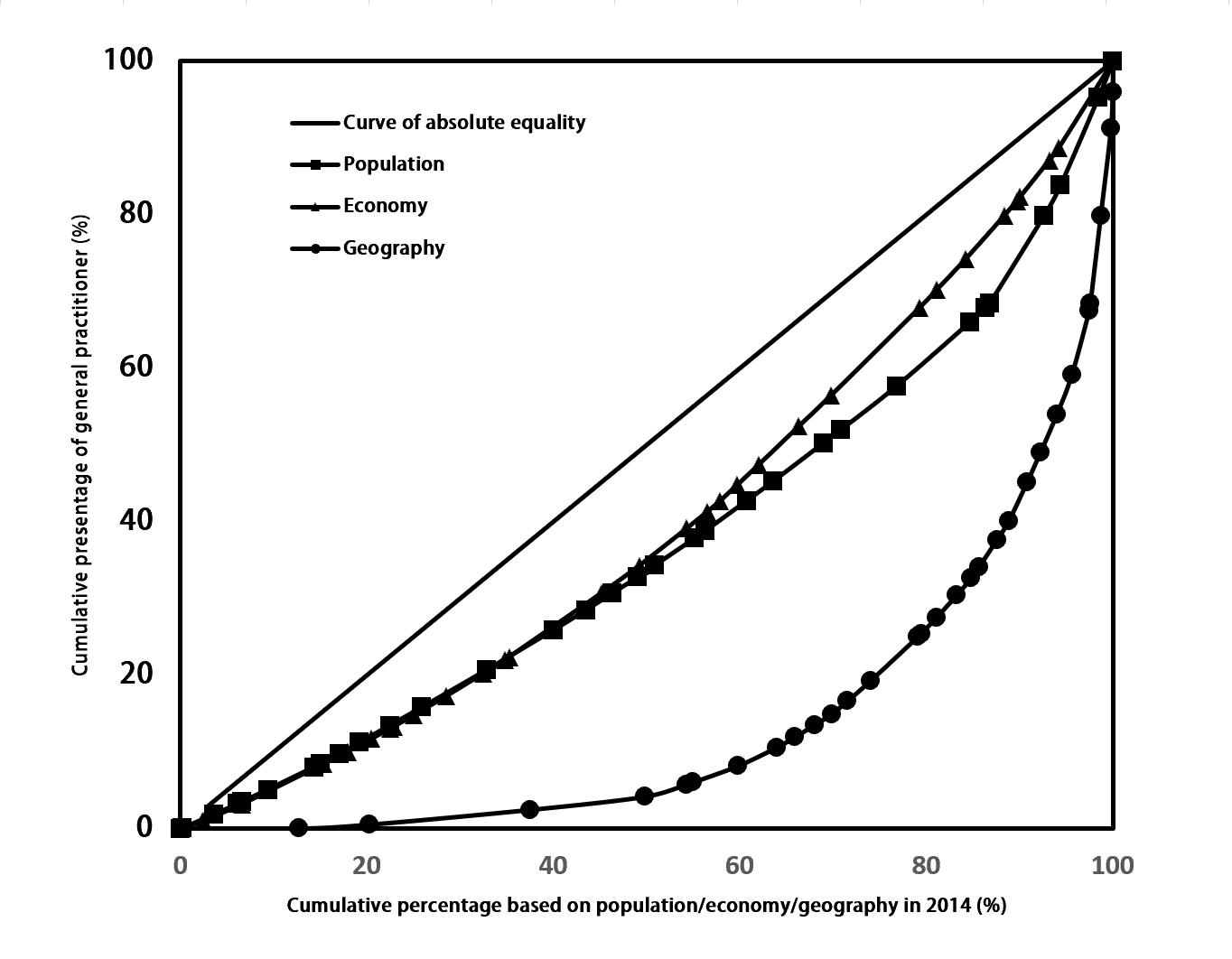

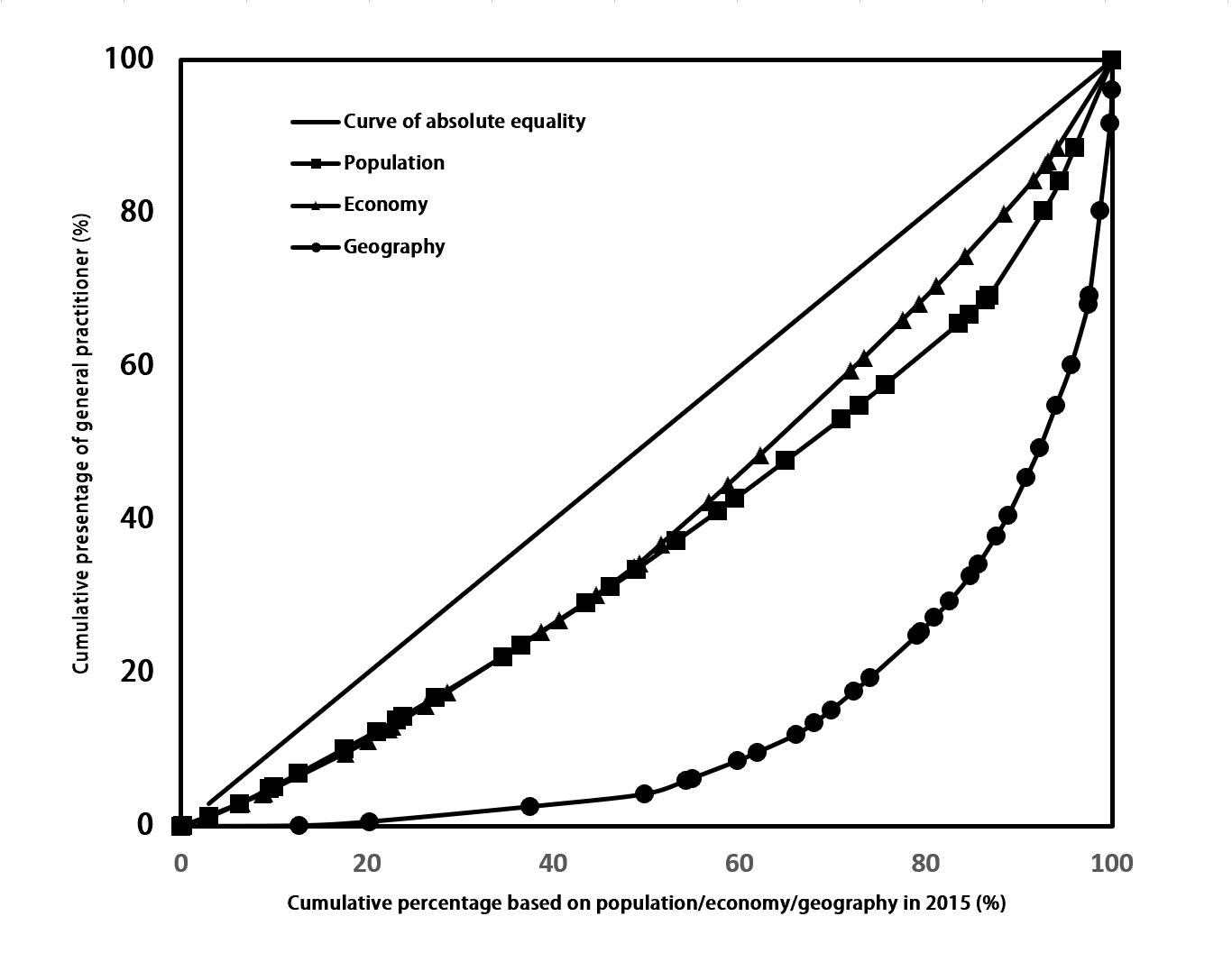


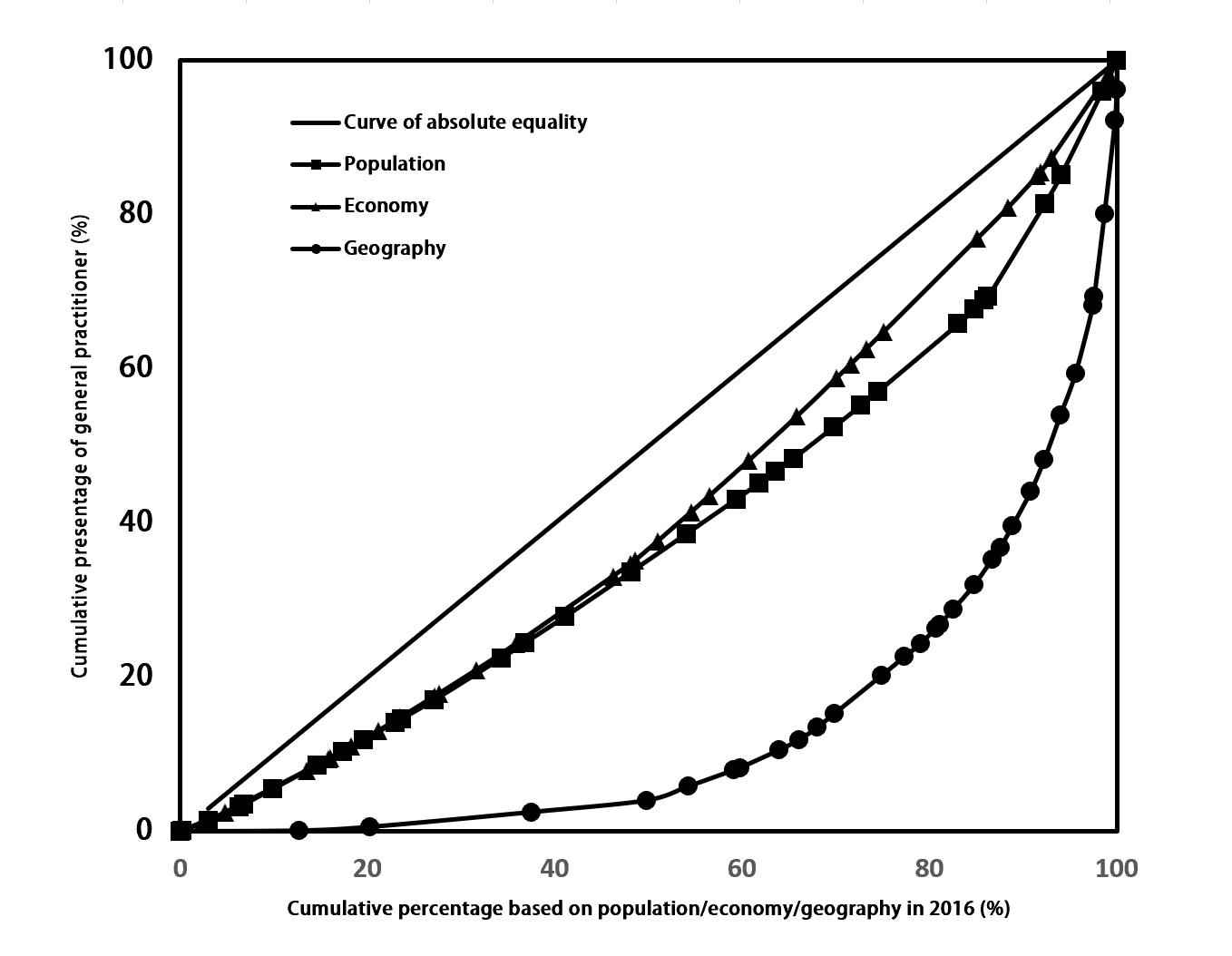


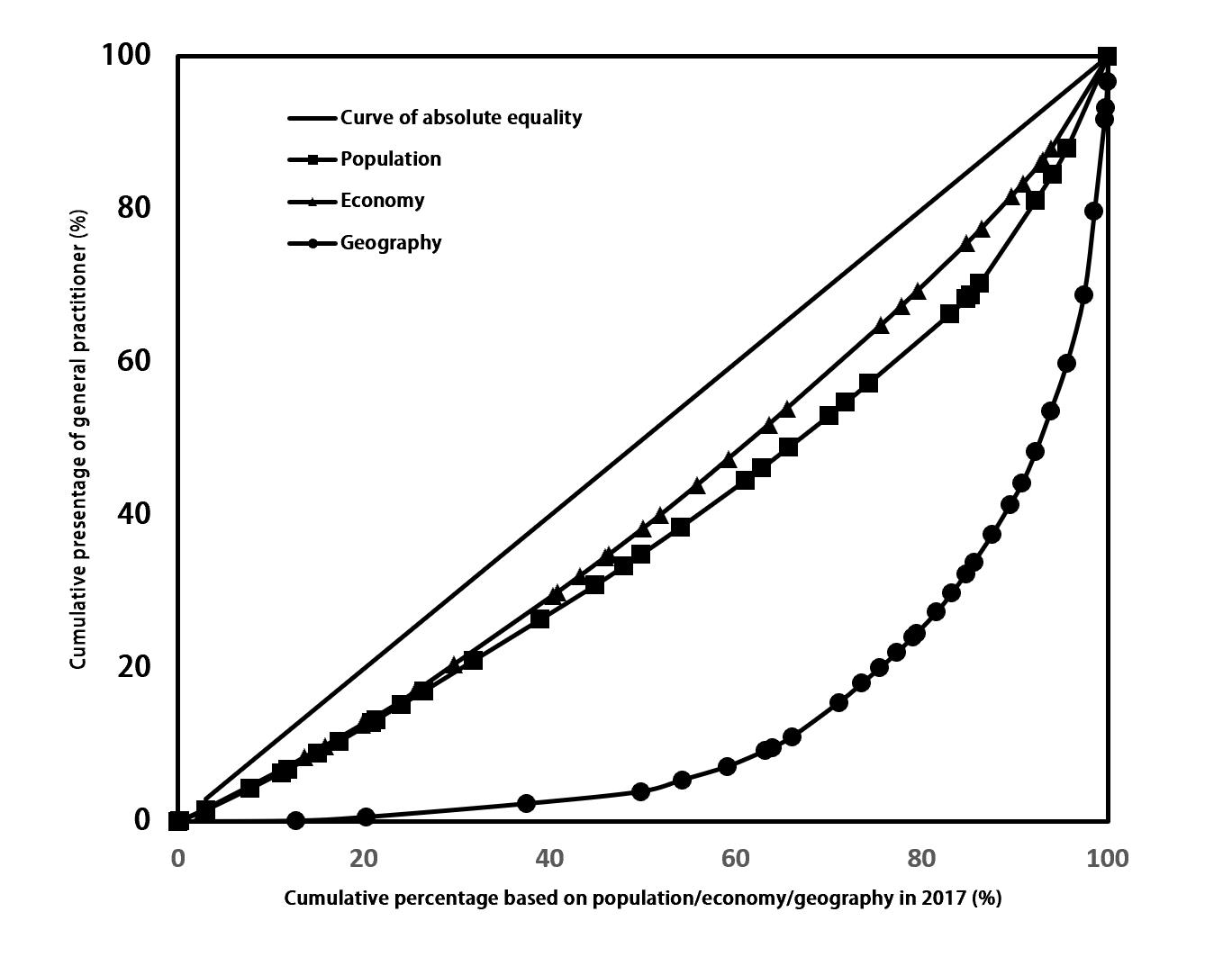


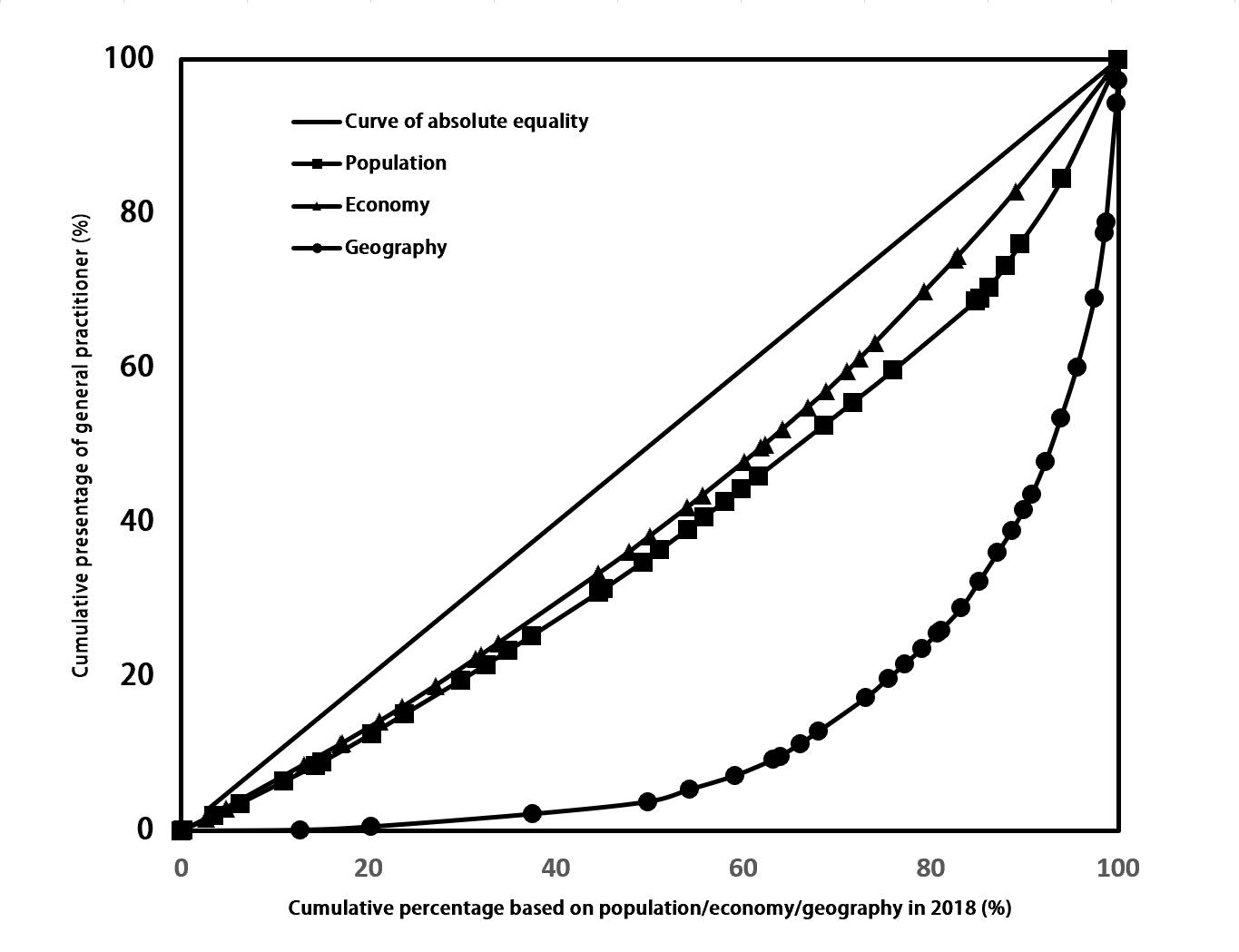

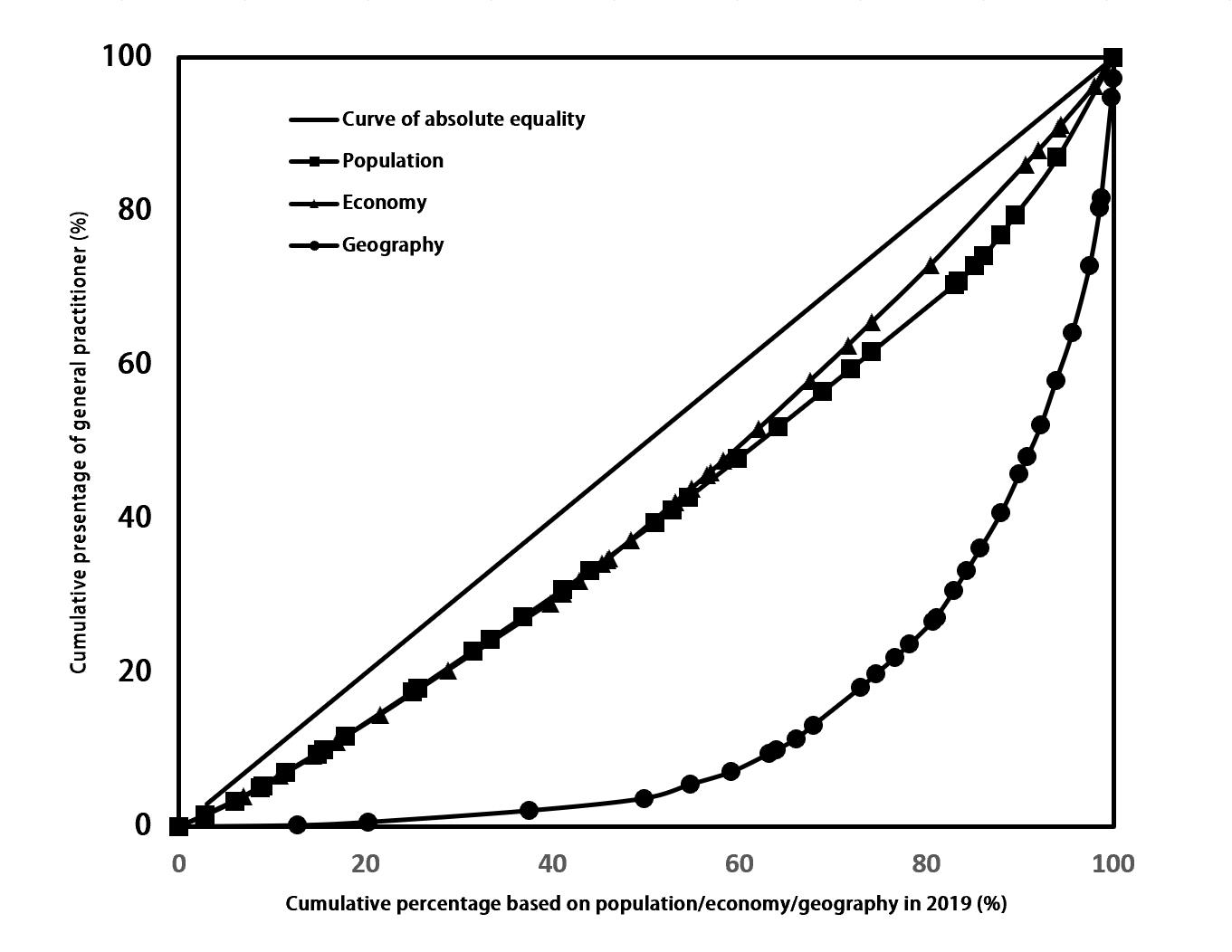


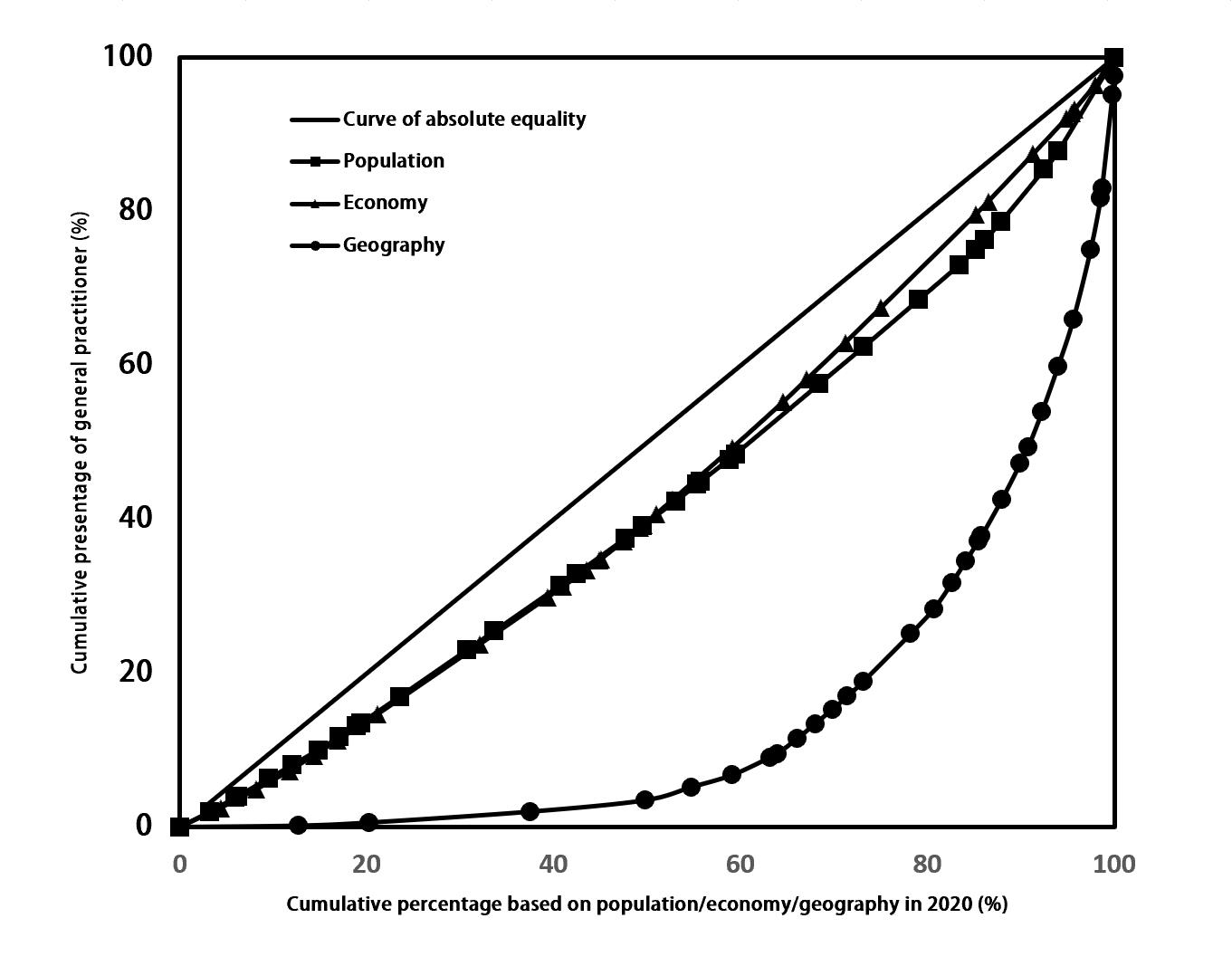


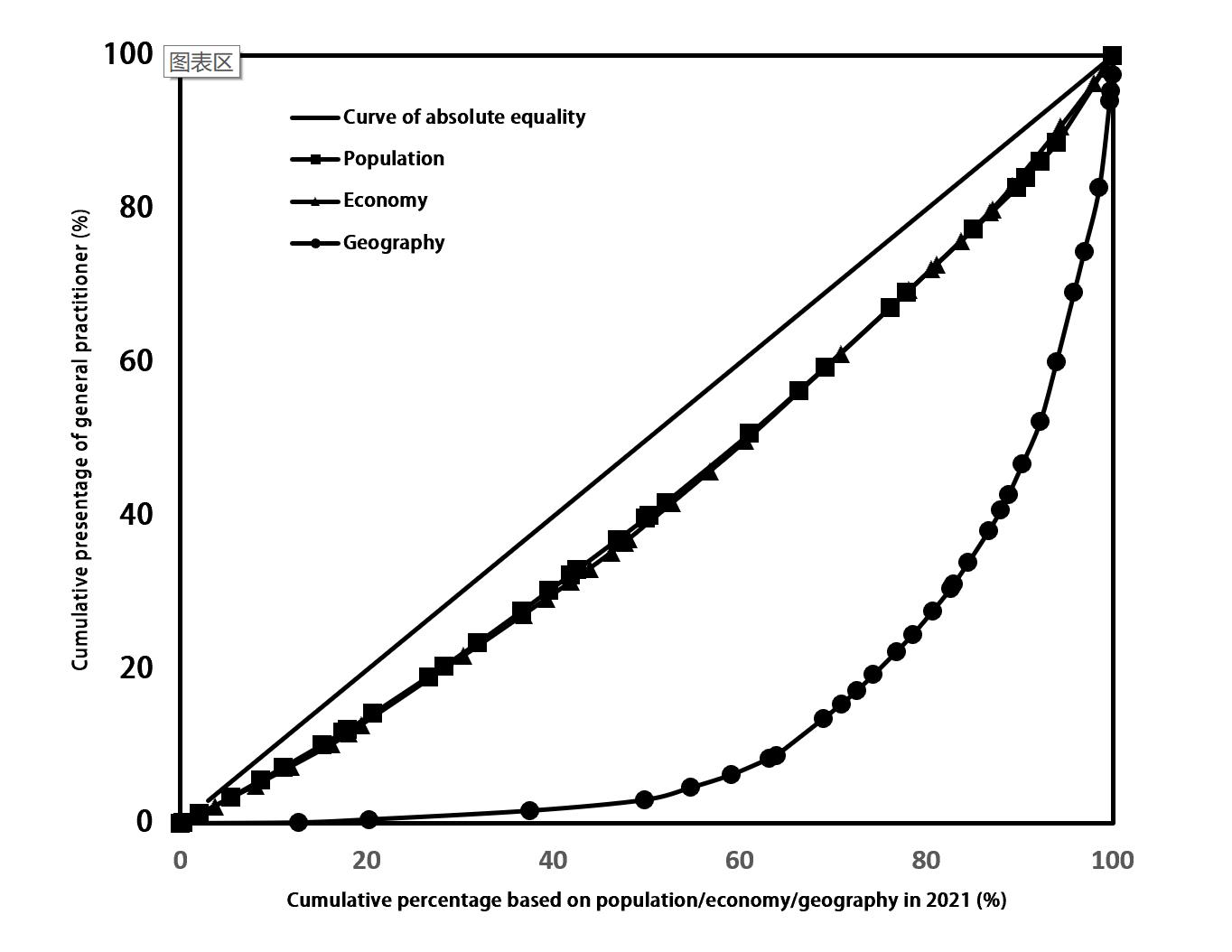


**Figure S1** Lorenz curve of Chinese GPs in 2012-2021

**Table S5** Agglomeration degree in 2012-2021

| **Region** | **Dimension** | **2012** | **2013** | **2014** | **2015** | **2016** | **2017** | **2018** | **2019** | **2020** | **2021** |
| --- | --- | --- | --- | --- | --- | --- | --- | --- | --- | --- | --- |
| Eastern region | HRAD | 5.416 | 5.198 | 5.032 | 4.938 | 4.991 | 4.942 | 4.942 | 4.713 | 4.553 | 4.618 |
|  | HRAD/PAD | 1.460 | 1.399 | 1.354 | 1.328 | 1.311 | 1.294 | 1.290 | 1.228 | 1.181 | 1.196 |
| Beijing | HRAD | 43.553 | 34.159 | 27.991 | 25.759 | 23.615 | 19.977 | 16.866 | 14.917 | 14.257 | 12.572 |
|  | HRAD/PAD | 4.828 | 3.724 | 3.016 | 2.768 | 2.545 | 2.166 | 1.837 | 1.632 | 1.563 | 1.379 |
| Tianjin | HRAD | 8.010 | 7.876 | 7.548 | 9.128 | 9.231 | 11.914 | 10.764 | 10.049 | 9.923 | 10.370 |
|  | HRAD/PAD | 0.951 | 0.903 | 0.844 | 1.007 | 1.107 | 1.471 | 1.360 | 1.272 | 1.256 | 1.327 |
| Hebei | HRAD | 1.631 | 2.371 | 2.565 | 2.523 | 2.294 | 2.032 | 1.875 | 2.585 | 2.382 | 2.878 |
|  | HRAD/PAD | 0.588 | 0.855 | 0.923 | 0.909 | 0.843 | 0.748 | 0.691 | 0.953 | 0.878 | 1.063 |
| Liaoning | HRAD | 1.988 | 1.595 | 1.446 | 1.269 | 1.325 | 1.640 | 1.926 | 1.963 | 1.902 | 1.811 |
|  | HRAD/PAD | 0.924 | 0.745 | 0.679 | 0.601 | 0.645 | 0.805 | 0.953 | 0.978 | 0.954 | 0.914 |
| Shanghai | HRAD | 74.167 | 62.627 | 61.379 | 59.619 | 58.292 | 51.399 | 42.756 | 41.584 | 36.956 | 37.546 |
|  | HRAD/PAD | 2.746 | 2.297 | 2.253 | 2.212 | 2.147 | 1.904 | 1.584 | 1.543 | 1.369 | 1.391 |
| Jiangsu | HRAD | 12.338 | 10.905 | 10.287 | 9.932 | 10.819 | 9.811 | 13.917 | 11.722 | 10.914 | 10.220 |
|  | HRAD/PAD | 2.336 | 2.070 | 1.958 | 1.899 | 1.996 | 1.811 | 2.572 | 2.168 | 2.019 | 1.885 |
| Zhejiang | HRAD | 10.193 | 10.698 | 10.395 | 10.473 | 9.862 | 11.013 | 7.707 | 6.858 | 6.174 | 4.925 |
|  | HRAD/PAD | 2.746 | 2.887 | 2.815 | 2.837 | 2.471 | 2.731 | 1.887 | 1.658 | 1.473 | 1.163 |
| Fujian | HRAD | 1.836 | 1.941 | 1.941 | 2.110 | 2.151 | 2.121 | 2.060 | 1.949 | 1.929 | 2.081 |
|  | HRAD/PAD | 0.850 | 0.897 | 0.894 | 0.970 | 0.958 | 0.938 | 0.906 | 0.854 | 0.841 | 0.902 |
| Shandong | HRAD | 3.817 | 3.277 | 3.214 | 3.253 | 3.365 | 3.320 | 3.491 | 3.564 | 3.746 | 5.109 |
|  | HRAD/PAD | 0.859 | 0.738 | 0.723 | 0.732 | 0.758 | 0.748 | 0.786 | 0.803 | 0.840 | 1.145 |
| Guangdong | HRAD | 3.879 | 4.336 | 4.476 | 4.252 | 4.704 | 4.820 | 4.801 | 4.694 | 4.877 | 4.812 |
|  | HRAD/PAD | 0.920 | 1.029 | 1.060 | 1.002 | 1.024 | 1.035 | 1.017 | 0.987 | 1.016 | 0.998 |
| Hainan | HRAD | 1.044 | 1.085 | 1.148 | 1.263 | 1.284 | 1.221 | 1.193 | 1.458 | 1.940 | 1.786 |
|  | HRAD/PAD | 0.583 | 0.604 | 0.636 | 0.698 | 0.685 | 0.645 | 0.626 | 0.758 | 0.993 | 0.907 |
| Middle region | HRAD | 1.153 | 1.164 | 1.290 | 1.372 | 1.363 | 1.429 | 1.392 | 1.483 | 1.484 | 1.493 |
|  | HRAD/PAD | 0.641 | 0.648 | 0.719 | 0.765 | 0.784 | 0.826 | 0.809 | 0.865 | 0.873 | 0.880 |
| Shanxi | HRAD | 1.430 | 1.250 | 1.289 | 1.309 | 1.228 | 1.551 | 1.188 | 1.098 | 1.058 | 1.052 |
|  | HRAD/PAD | 0.868 | 0.759 | 0.783 | 0.796 | 0.790 | 1.004 | 0.774 | 0.719 | 0.695 | 0.694 |
| Jilin | HRAD | 0.577 | 0.594 | 0.685 | 0.788 | 0.832 | 1.044 | 0.827 | 1.062 | 1.005 | 0.978 |
|  | HRAD/PAD | 0.550 | 0.569 | 0.659 | 0.763 | 0.876 | 1.123 | 0.908 | 1.187 | 1.149 | 1.130 |
| Heilongjiang | HRAD | 0.386 | 0.405 | 0.440 | 0.467 | 0.434 | 0.362 | 0.372 | 0.368 | 0.346 | 0.324 |
|  | HRAD/PAD | 0.666 | 0.702 | 0.768 | 0.824 | 0.855 | 0.731 | 0.770 | 0.781 | 0.755 | 0.717 |
| Anhui | HRAD | 1.999 | 2.042 | 2.716 | 2.684 | 2.838 | 2.839 | 2.878 | 2.848 | 3.113 | 2.705 |
|  | HRAD/PAD | 0.654 | 0.667 | 0.884 | 0.871 | 0.950 | 0.952 | 0.966 | 0.957 | 1.045 | 0.907 |
| Jiangxi | HRAD | 1.094 | 0.964 | 1.010 | 1.016 | 1.006 | 1.204 | 1.051 | 1.061 | 1.134 | 1.278 |
|  | HRAD/PAD | 0.567 | 0.500 | 0.525 | 0.528 | 0.538 | 0.646 | 0.566 | 0.573 | 0.613 | 0.691 |
| Henan | HRAD | 2.482 | 2.549 | 2.807 | 3.166 | 3.348 | 3.555 | 3.831 | 3.598 | 3.438 | 4.490 |
|  | HRAD/PAD | 0.616 | 0.636 | 0.702 | 0.793 | 0.825 | 0.876 | 0.944 | 0.887 | 0.845 | 1.110 |
| Hubei | HRAD | 1.772 | 1.797 | 1.829 | 1.915 | 1.741 | 1.840 | 1.824 | 1.826 | 1.756 | 1.505 |
|  | HRAD/PAD | 0.797 | 0.810 | 0.827 | 0.866 | 0.793 | 0.840 | 0.834 | 0.837 | 0.831 | 0.702 |
| Hunan | HRAD | 1.070 | 1.228 | 1.333 | 1.476 | 1.418 | 1.268 | 1.303 | 2.089 | 2.182 | 1.879 |
|  | HRAD/PAD | 0.477 | 0.547 | 0.592 | 0.656 | 0.654 | 0.587 | 0.606 | 0.974 | 1.017 | 0.880 |
| Western region | HRAD | 0.271 | 0.302 | 0.297 | 0.292 | 0.286 | 0.277 | 0.286 | 0.300 | 0.325 | 0.312 |
|  | HRAD/PAD | 0.714 | 0.798 | 0.784 | 0.769 | 0.755 | 0.731 | 0.754 | 0.789 | 0.852 | 0.821 |
| Inner Mongolia | HRAD | 0.125 | 0.133 | 0.139 | 0.133 | 0.124 | 0.128 | 0.129 | 0.129 | 0.120 | 0.114 |
|  | HRAD/PAD | 0.828 | 0.885 | 0.926 | 0.893 | 0.867 | 0.906 | 0.918 | 0.926 | 0.867 | 0.825 |
| Guangxi | HRAD | 1.140 | 1.126 | 1.064 | 1.004 | 0.990 | 1.007 | 1.046 | 1.185 | 1.305 | 1.221 |
|  | HRAD/PAD | 0.809 | 0.797 | 0.752 | 0.708 | 0.699 | 0.707 | 0.731 | 0.825 | 0.904 | 0.843 |
| Chongqing | HRAD | 1.739 | 1.758 | 1.712 | 1.781 | 1.749 | 1.789 | 2.405 | 2.600 | 2.509 | 2.406 |
|  | HRAD/PAD | 0.680 | 0.686 | 0.667 | 0.692 | 0.668 | 0.680 | 0.912 | 0.982 | 0.943 | 0.903 |
| Sichuan | HRAD | 0.843 | 1.224 | 1.128 | 1.093 | 0.983 | 0.890 | 0.861 | 0.969 | 1.223 | 0.947 |
|  | HRAD/PAD | 0.709 | 1.032 | 0.952 | 0.921 | 0.835 | 0.757 | 0.732 | 0.824 | 1.039 | 0.805 |
| Guizhou | HRAD | 0.514 | 0.568 | 0.766 | 0.912 | 0.972 | 1.085 | 1.105 | 0.969 | 1.013 | 1.166 |
|  | HRAD/PAD | 0.364 | 0.402 | 0.544 | 0.648 | 0.657 | 0.729 | 0.742 | 0.648 | 0.677 | 0.781 |
| Yunnan | HRAD | 0.715 | 0.716 | 0.582 | 0.556 | 0.554 | 0.508 | 0.505 | 0.590 | 0.567 | 0.520 |
|  | HRAD/PAD | 0.846 | 0.847 | 0.688 | 0.657 | 0.673 | 0.619 | 0.617 | 0.721 | 0.693 | 0.640 |
| Tibet | HRAD | 0.002 | 0.004 | 0.005 | 0.007 | 0.008 | 0.008 | 0.009 | 0.014 | 0.014 | 0.008 |
|  | HRAD/PAD | 0.136 | 0.200 | 0.271 | 0.361 | 0.395 | 0.391 | 0.452 | 0.686 | 0.688 | 0.414 |
| Shaanxi | HRAD | 0.779 | 0.637 | 0.752 | 0.528 | 0.614 | 0.664 | 0.756 | 0.681 | 0.929 | 1.429 |
|  | HRAD/PAD | 0.597 | 0.489 | 0.579 | 0.407 | 0.470 | 0.507 | 0.576 | 0.518 | 0.706 | 1.087 |
| Gansu | HRAD | 0.286 | 0.328 | 0.355 | 0.397 | 0.408 | 0.342 | 0.354 | 0.372 | 0.361 | 0.386 |
|  | HRAD/PAD | 0.661 | 0.760 | 0.826 | 0.926 | 0.995 | 0.839 | 0.874 | 0.921 | 0.899 | 0.967 |
| Qinghai | HRAD | 0.056 | 0.070 | 0.068 | 0.068 | 0.063 | 0.065 | 0.057 | 0.055 | 0.053 | 0.052 |
|  | HRAD/PAD | 0.990 | 1.221 | 1.193 | 1.188 | 1.134 | 1.161 | 1.018 | 0.990 | 0.945 | 0.921 |
| Ningxia | HRAD | 0.344 | 0.391 | 0.396 | 0.435 | 0.454 | 0.532 | 0.601 | 0.596 | 0.582 | 0.543 |
|  | HRAD/PAD | 0.493 | 0.558 | 0.562 | 0.615 | 0.626 | 0.726 | 0.819 | 0.807 | 0.784 | 0.728 |
| Xinjiang | HRAD | 0.101 | 0.108 | 0.112 | 0.114 | 0.111 | 0.102 | 0.095 | 0.087 | 0.082 | 0.066 |
|  | HRAD/PAD | 1.058 | 1.118 | 1.142 | 1.141 | 1.101 | 0.989 | 0.918 | 0.825 | 0.775 | 0.625 |
